# Co-existence of chronic spontaneous urticaria with atopic dermatitis: clinical and immunological perspectives

**DOI:** 10.3389/falgy.2025.1681375

**Published:** 2025-11-24

**Authors:** Elias Toubi, Raeda Mubariki, Zahava Vadasz

**Affiliations:** 1 The Allergy Out-Patient Clinic, The Holy Family Hospital, Nazareth, Israel; 2 The Clinical Immunology and Allergy, The Bnai-Zion Medical Center, Haifa, Israel; 3 The Rappaport Faculty of Medicine, Technion – Israel Institute of Technology, Haifa, Israel

**Keywords:** CSU, atopic dermatitis, asthma, total IgE, anti-TPO

## Abstract

**Background:**

Chronic Spontaneous Urticaria (CSU) and Atopic Dermatitis (AD) are both immune-mediated inflammatory skin disorders that often co-exist with other atopic conditions such as asthma and allergic rhinitis. Their shared immunopathological pathways raise the question of a possible interrelationship.

**Objective:**

To evaluate the prevalence, clinical features, and immunological profiles of AD in patients with CSU and to explore implications for diagnosis and treatment.

**Methods:**

425 CSU patients treated in Northern Israel between 2021 and 2024, were retrospectively analyzed. Disease activity was assessed using the Urticaria Activity Score-7 (UAS7) and Investigators' Global Assessment (IGA) for AD. The prevalence of asthma, total serum IgE levels, and therapeutic responses were evaluated.

**Results:**

Among the 425 CSU patients, 42 (10%) were also diagnosed with AD. Co-morbid patients had a higher frequency of asthma (40%) and high total IgE levels (67%) compared to CSU-only patients. A substantial subset of co-morbid cases required biologic treatments with Dupilumab, offering benefit in AD-dominant cases unresponsive to Omalizumab. Severe CSU was more prevalent in the CSU + AD group (though the prevalence was not statistically significant).

**Conclusion:**

CSU and AD frequently co-exist, likely due to overlapping T-cell–mediated immunopathogenic mechanisms. High total IgE and asthma comorbidity may indicate an underlying AD component in CSU patients. Recognition of this overlap is essential for appropriate therapeutic decision-making, including potential escalation to biologic agents targeting T-cell cytokine pathways.

## Introduction

Atopy is frequently presented as a collection of comorbid conditions. With this in mind, the co-existence of autoimmune skin diseases, namely, alopecia areata (AA) or vitiligo with chronic spontaneous urticarial (CSU) and atopic dermatitis (AD) was previously reported. Pooled analysis of three studies found higher odds ratios (OR) of AD patients with vitiligo and in four other studies, higher OR of AD was recorded in patients with alopecia totalis compared with those with patch alopecia (OR, 1.22; 95% CI, 1.01–1.48, *p* < 0.04)) (1). In another study, the OR of having CSU with AA was 6.15 (4.06–9.32; *p* < 0.001). In addition, among AA patients, there was higher prevalence of AD, allergic rhinitis and asthma than in the control group (2). The association of CSU with many atopic diseases was assessed in a nationwide population-based study including; 9,332 patients with CSU and 37,328 controls, matched for age and sex. In this study, CSU was strongly associated with many immune-mediated inflammatory diseases such as allergic rhinitis, asthma, and AD (3). Consistent with this, medical records of 1.108.833 adolescents were reviewed in another nationwide population study. 6,617 (0.6%) of those adolescents, suffered from CSU. In this study, CSU patients were significantly more likely to have allergic diseases including allergic rhinitis (OR, 2.9, 95% CI, 2.71–3.11), and AD (OR, 2.35, 95% CI, 2.03–2.72) (4). In a later study, a systemic review and meta-analysis was performed to assess the association of CSU, AD and asthma. In thirty-eight studies, pooled point prevalence of AD in CSU patients was 7% (5%–11%, 12 = 99%) and of asthma was 12% (9%–15%, 12 = 100%). Pooled point prevalences of atopic disorders among patients with CSU were comparable to the general population. However, studies that compared the prevalence of atopic disorders in CSU with controls from the same population found an increased risk of atopic disorders in CSU patients (5). CSU can serve as a potential factor or further potential risk factor for progression of the atopic march. The importance of understanding and defining the association between immune-mediated skin diseases is necessary because the treatment of one condition influences the others, and the development of one may be followed by the development of another. In our study, we aimed to assess the prevalence and characteristics of AD in a large cohort of CSU patients followed in our outpatient clinics in the North of Israel.

## Patients and methods

Four hundred and twenty-five CSU patients (295 women, age 19–64 years, and 130 men, age 17–69 years) were followed in our two large outpatient clinics in the North of Israel between 2021 and 2024. CSU disease activity was defined using Urticaria Activity Score-7 (UAS7) (6). CSU disease duration was between 1 and 5 years). During the whole period of follow-up, 42 patients (10%) presented with classical co-morbid episodes of AD (23 women, age 32–59 years; and 19 men, age 41–64 years). Atopic dermatitis was diagnosed based on the Hanifin and Rajka criteria (7) and modified by Wollenberg et al. (8). The commonly used Investigators' Global Assessment (IGA) defined AD disease activity (9). Both groups: (CSU-383 patients and CSU + AD-42 patients) were assessed for the co-presence of asthma, and the level for serum total IgE and IgG anti-TPO. High total IgE levels were defined as >150 IU/ml and high IgG anti-TPO as >35 IU/ml. In addition, treatment decisions were applied according to the individual clinical status.

### Statistical analysis

Statistical comparisons were conducted between CSU and CSU + AD patients. Severity distributions were compared using the Chi-square test of independence. Due to small expected counts in some cells, Fisher's exact test was used for binary variables (asthma and IgE). A significance level of 0.05 was applied to all tests. Statistical analyses were performed using GraphPad Prism.

## Results

### Characteristics of CSU patients

**Figure 1:**
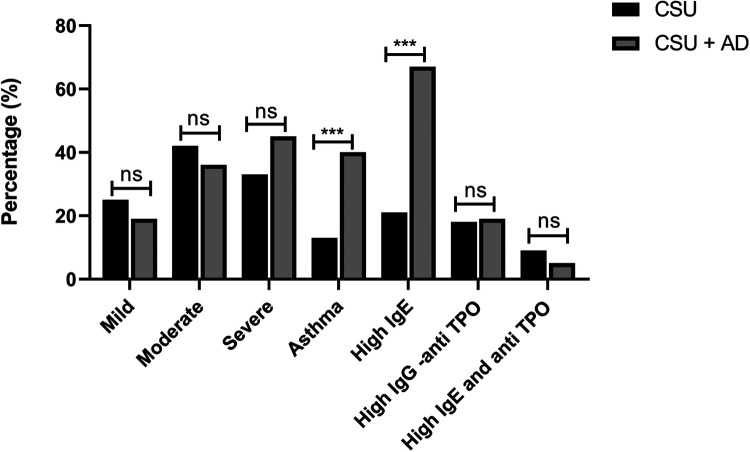
Presents the percentages of the following categorical variables: severity (mild, moderate, severe), presence of asthma, high total IgE and IgG anti-TPO, compared between CSU and CSU + AD. ns. not significant, ***p-value < 0.001.

Ninety-seven of 383 CSU patients (25%) were defined as having a mild disease (UAS <12 points) and were adequately controlled with antihistamines only. Of these, 12 patients (12%) had high levels of total IgE (170–210 IU/ml) and nine (10%) had high IgG anti-TPO. Hundred and sixty (42%) were considered moderate (UAS7-12–30 points) and were reasonably controlled with high doses of anti-histamines and in some montelukast was added. Among these, high levels of total IgE (180–310 IU/ml) was recorded in 35 (22%) patients, and 31 (19%) had high IgG anti-TPO. Hundred and twenty-six (33%) patients were defined as having severe CSU (UAS 7 > 30 points). UAS7 was assessed in all CSU patients at baseline before the initiation of high doses of antihistamines or omalizumab), Severe CSU patients were effectively treated with omalizumab, achieving a clinical response between good to excellent. Of these, 34 (27%) patients had high levels of total IgE (220–390 IU/ml) and 31 (26%) had high IgG anti-TPO. Both high total IgE and high anti-TPO were found to be in 34 (9%) of patients Bronchial asthma was diagnosed in 49 (13%) of all CSU patients, mostly controlled with standard doses of inhaled steroids. Among these high total IgE was noticed in 14/49 (28%). *Characteristics of patients with CSU and AD co-morbidity:* Of all 42 patients eight patients (19%) were defined as having mild CSU, fifteen (36%) were moderate and 19 (45%) had severe CSU and were treated with omalizumab. High total IgE was recorded in 28/42 patients (67%) and high IgG anti-TPO in 19% of patients. Both high total IgE and high anti-TPO were found in only 2 (5%) of these patients. Bronchial asthma was co-present in 17 (40%) patients (Table 1 and Figure 1). Of these high total IgE was recorded in 9/17 (53%). Of all 42 patients with CSU and AD patients, 23 (55%) were classified as mild AD (IGA1) and were well controlled with periodic topical steroids and antihistamines. The other 19 patients in whom AD was co-present with CSU, the disease was classified as moderate-severe (IGA 2–3), and required a systemic therapeutic approach. Of these 10/19 (53%) patients, AD was poorly controlled and was considered to be the dominant problem. The possibility of switching omalizumab to another biological treatment was raised. In three patients, due to their high AD disease severity (IGA 3–4), the treatment was switched from omalizumab to dupilumab. In this respect, dupilumab is a well-approved and highly beneficial biological drug for severe AD. In many recent studies, dupilumab was also shown to be a possible option for treating CSU, mainly those who were resistant to omalizumab. In our three patients, IGA score was decreased to 1–2 points, and CSU activity remained stable with UAS7 around 6–8 points.

**Table 1:** Characteristics of CSU patients: the table describes disease severity (mild, moderate and severe), asthma comorbidity, high total levels of IgE, number of patients with high IgG anti-TPO and high levels of IgE and anti-TPO simultaneously.

CSU characteristic	CSU *n* = 383	CSU + AD *n* = 42	*P* value
Mild	97 (25%)	8 (19%)	ns
Moderate	160 (42%)	15 (36%)	ns
Severe	126 (33%)	19 (45%)	ns
Asthma	49 (13%)	17 (40%)	***
High total IgE	81 (21%)	28 (67%)	***
High IgG anti-TPO	71 (18%)	8 (19%)	ns
High both IgE and anti-TPO	34 (9%)	2 (5%)	ns

ns, not significant.

*** *P* value < 0.0001.

## Discussion

Chronic Spontaneous Urticaria (CSU) and Atopic Dermatitis (AD) are prevalent immune-mediated inflammatory skin diseases that often coexist with allergic rhinitis, asthma, and food allergies. The shared immune features of these disorders include T cell activation, mast cell involvement, total IgE, and elevated inflammatory mediators such as IL-4, IL-13, IL-17, and IL-31. In CSU, both systemic and local immune responses involve Th1, Th2, Th17, and Th22 cells, correlating with disease severity. These cytokines and T cell infiltrates contribute to skin inflammation, pruritus, and tissue remodeling (10, 11). Similarly, AD exhibits a shift from Th2 to Th1 dominance in chronic stages, with overlapping cytokine profiles, underscoring a common immunological basis. The release of TNF-α, IFN-γ, IL-17, and other mediators such as MMP-9 by mast cells in both CSU and AD, contribute to T-cell recruitment and extravasation in lesional skin (12). The above pro-inflammatory cytokines are major players in the development of skin eczematous inflammation, itch and keratinocyte apoptosis. Autoimmunity appears to play a central role in both diseases. IgE antibodies and autoreactive T cells directed against skin-specific antigens are well documented in CSU but have been also implicated in AD. These findings support the notion that CSU and AD may share autoimmune triggers, which might explain their possible co-occurrence (13, 14). Our study demonstrated that CSU patients with concurrent AD exhibited more severe CSU (though of no statistical significance) and more frequently had asthma and high serum IgE levels, suggesting both CSU and AD to be frequent conditions during the atopic march. Yet, as it is shown in many studies, these patients have increased infiltrates of Th1, Th2 and Th17 cells, significantly higher than that of CSU-only patients. This clinical observation aligns with the emerging understanding that in patients with high T-cell activity or coexisting atopic conditions, T-cell targeted therapies may be more effective. Notably, these patients, especially those of poor response to Omalizumab necessitated the targeting of T cells, namely, the switch to Dupilumab, and in some cases the consideration of applying anti-IL-17 or JAK-inhibitors. It also supports the potential role of personalized medicine, guided by biomarkers in managing complex cases. Biomarkers such as total IgE, autologous serum test, and cytokines such as IL-4, IL-13 and IL-17, may serve beyond the diagnosis of skin diseases such as CSU and AD, offering insights into therapeutic monitoring. However, most of these biomarkers hold more scientific than clinical values and reveal limited specificity (15). A multidisciplinary approach is crucial for optimizing outcomes in these patients. Larger cohorts and long-term follow-up studies are required to validate these findings and better define the CSU-AD overlap condition. The growing use of biologics in dermatology emphasizes the need for individualized treatment strategies informed by immunological markers and clinical presentation.

## Data Availability

The raw data supporting the conclusions of this article will be made available by the authors, without undue reservation.
